# A Cross-Sectional Survey on Parasites of Chickens in Selected Villages in the Subhumid Zones of South-Eastern Nigeria

**DOI:** 10.1155/2010/141824

**Published:** 2010-07-27

**Authors:** P. A. Nnadi, S. O. George

**Affiliations:** Department of Animal Health and Production, Faculty of Veterinary Medicine, University of Nigeria, Nsukka, Nigeria

## Abstract

A study was carried out to identify and estimate the prevalence of ecto- and endoparasites of village chicken between April and July 2008 in three local councils of Enugu state, Nigeria. A total of 1038 chickens comprising of 468 chicks, 207 growers and 363 adults were examined during the house to house survey for ectoparasites, gastrointestinal helminths and coccidia infections. Our finding showed that 41% were infected with ectoparasites with lice, fleas, and mites having prevalence rates of 62.2%, 35.7% and 2.1%, respectively. Helminths and coccidia had prevalence of 35.5% each. Among the helminths *Ascaridia, galli* was the most dominant species (17.2%). Generally, there was a significantly higher helminth infestation relative to the ectoparasites (*P* < .05), high prevalence of mixed infections and absence of tick infestation. Parasitism could be big constraint to production in the study area and we recommend a sustainable control strategy.

## 1. Introduction

The Poultry industry occupies an important position in the provision of animal protein (meat and egg) to man and generally plays a vital role in the national economy as a revenue provider. Poultry is one of the most intensively reared of the domesticated species and one of the most developed and profitable animal production enterprises [1]. Its importance in national economies of developing countries and its role in improving the nutritional status and income of many small farmers and those with small land holdings as well as landless has been recognised by various scholars and rural development agencies in the last two decades [2–4]. 

Poultry production in Africa and parts of Asia is still distinctively divided into commercialized and village enterprise subsector, each with its peculiarities. The former comprises of strains specifically developed on the basis of primary products into parent stocks, layers, and broilers each with its specialized equipments and management approach. The latter however, consists of indigenous domestic fowls *(Gallus domesticus)* variously referred to as local or rural chickens, backyard poultry or village chickens, and or free range chickens. These refer to breeds∖strains∖ecotypes with no improvement history [5] and chickens indigenous to the particular locality they are found. These constitute a rich genetic resource base for any future genetic improvement and production of strains adaptable to the tropics [6]. 

In most African countries, backyard poultry account for more than 60% of the total national poultry flocks accorded an asset value of more than 5.75 billion US$ [7]. It is estimated that these provide 12 kg of poultry needs per inhabitants per year whereas cattle provides 5.3 kg [8]. This means that in comparison, poultry meat is more available to the people more than beef. In Nigeria, the population of poultry is estimated to be about 140 million with backyard poultry constituting about 60%, thus, the most important form of poultry production [9]. Flock sizes range from 5–50. Main utilities include home consumption (meat and eggs), and other social obligations [4, 10, 11]

Village chicken production is constrained by many extrinsic factors among which malnutrition, poor management and the absence of biosecurity are outstanding. Losses have also been attributed to limited housing and veterinary care services. Furthermore, poor genetic potential due to lack of selection and predation are also potential threats to productivity [12]. 

Parasitism ranks high among factors that threaten village chicken production [13]. The authors reported that mortality due to parasitic diseases was higher than those attributed to Newcastle disease, an acknowledged most endemic and mortality causing viral infection of poultry. Common poultry parasites range from lice, mites, fleas, ticks, and helminths to gnats and coccidia [14]. Parasitism causes reduced growth, egg production, emaciation, and anaemia as well as mortality [15–19]. Moreover, some of the ectoparasites, especially tick and mites, are vectors of other poultry diseases such as pastuerellosis, Fowl pox, Newcastle disease, and possibly chlamydia [17, 20, 21]. In addition, the roles of poultry worms such as *Heterakis gallinarum* has been associated with the transmission of *Histomonas meleagridis* in turkeys and chicks [20, 21]. Moreover, it has been reported that parasitic infection or their concurrent infections result in immunosuppression, especially in response to vaccines against some poultry diseases. Studies in other countries had shown that the prevalence of parasitic infestations in village chicken flocks is close to 100%, and in most cases individual birds' habour more than one parasite type [22]. In Zambia, [23] reported helminth prevalence at 95.2%, while in Tanzania, [24] reported 52%. In Northern Nigeria, a study showed that the prevalence rate of helminth infection is about 70% [25]. Reports also exist on the prevalence of coccidiosis of village chicken [26]. 

Currently, there is a paucity of information regarding the prevalence of ecto- and endoparasites of local chickens in the study area. There is also the need to constantly assess the status of village chicken production constraints and the dynamic of their interactions. In addition, as cofactors in other poultry diseases, the knowledge of their prevalence is essential in understanding the epidemiology of such diseases and the design of their appropriate control measures. The current study was designed to investigate the prevalence of ecto- and endoparasites of village chickens in the subhumid tropics of South Eastern Nigeria.

## 2. Materials and Methods

### 2.1. Method of Sampling and Population Size

The study area comprised villages in three local Government councils within Nsukka Zone of Enugu State, Nigeria namely Igboeze South, Nsukka Urban and Udenu Local councils) with a subhumid tropical climate. Three villages lacking in contiguous boundaries were selected within each local council with an average of eight villages. Within each village comprising of about 100 households, 10 were randomly selected for sampling. A minimum of ten birds were again randomly sampled per household without consideration for age or sex. A total of one thousand and thirty eight chickens were involved in the survey. The sampling was done between the months of April and July 2008. Within the survey period, presurvey visits were made to the selected households for an agreement on the sampling dates and to provide them with a locally made basket for the restraint of the birds over night. During our interaction with the village chicken keepers, enquiries pertaining to mortality patterns among the various age groups, and the distribution of observable parasites were made. Information regarding the dynamics of flock size, number of eggs laid before incubation, percentage hatch, number successfully brooded, and number that attain adulthood were orally obtained from the poultry keepers.

### 2.2. Screening for Ectoparasites

Screening for ectoparasites involved a thorough examination of the body of the birds including the head, cloacal, brachial, ventral, and femoral areas. Those with parasites were identified and recorded. Also, samples of the observed parasites were removed with a thumb forceps or camel hair brush and transferred to a Petri dish containing 10% farmol saline. They were cleared with lactophenol and fixed on a microscopic slide using a little quantity of polyvinyl alcohol and lactophenol solution before detailed morphological examination and identification using a compound microscope [27, 28]. Following this, birds that were positive for ectoparasites were liberally dusted with an insecticide powder, Piff Paff^R^ (permetrine powder). Also, thorough examination of cracks and crevices within the sleeping areas of the chickens was carried out to ensure that those parasites with nocturnal activities are identified.

### 2.3. Faecal Collection and Analyses for Helminth Eggs and Coccidia Oocysts

For each of the birds, after thorough examination for ectoparasites, faecal samples were collected per cloaca where possible or with a spatula for freshly voided faeces. As it was not possible to collect faecal samples from all the birds examined for ectoparasites, where it was possible the sample was matched with the record of ectoparasitism. The faecal sample were put into sample bottles and identified appropriately. The samples were later processed in the laboratory using the salt floatation technique with saturated sodium chloride solution as the floating medium [29]. Identification of helminth eggs and coccidia oocysts was done using a standard microscope under ×10 objective magnification. This was a qualitative assessment. Thorough examination was made to separate strongyle eggs from those of cestodes. In some cases proglotides or whole worms were collected∖harvested with the faecal samples. Moreover, from each village, ten birds were selected randomly and paid for the purposes worm recovery and identification after necropsy in our laboratory. These were humanely slaughtered, eviscerated and the content of the gastrointestinal tract harvested, washed thoroughly for possible worm recovery and identification according to [28].

### 2.4. Statistics

Student's *t*-test was used to compare the prevalence rate between ecto- and endoparasites and comparing the incidence of the various ectoparasite species.

## 3. Results

### 3.1. Productivity Profile

Information from the village poultry keepers reveal that most hens lay between 6–18 eggs prior to natural incubation out of which hatchability varies between hens. Average hatchability is 70% with variability again on the number successfully brooded which varies between hens. Depending on season between 50–80% of the hatchlings are usually successfully brooded. Due to the undefined production objective, chicks are sold at any age and used for various purposes.

### 3.2. Prevalence of Ectoparasites

The result of this study showed that based on age strata the adults chickens were the most infested with ectoparasites (Table 1). They were followed by the growers and lastly the chicks. Also of the three common ectoparasites encountered, lice infestation was significantly higher than flea and mites while lice was higher than mites (*P* < .05). Generally, of the 1038 chicken population sampled, 427 representing 41.1% were infested by various species of ectoparasites. Table 2 below shows the prevalence of the various ectoparasite species.

### 3.3. Prevalence of Endoparasites

The result of the faecal analyses showed that of the 261 faecal samples collected, 186 (71.3%) of the samples were collectively positive for helminth eggs and coccidia oocysts. Moreover, it was observed that the two enteric parasites had equal prevalence of 35.5% each, representing 93 members of the sample population. Comparisons between endo- and ectoparasite prevalence indicated a statistically significant higher prevalence in favour of the former (*P* < .05). Moreover, among those positive for gastrointestinal helminthes, there were variations in the prevalence of the various helminth types in the population especially between the nematodes and cestodes as shown in the Table 3. Of the three hundred chickens necropsied for helminth recovery and identification, two hundred and ten, representing 70% of the sample population, were positive for various helminth species. Table 3 below shows the prevalence of the various helminth species in the sample population.

Furthermore, our study showed that parasitic infestations are usually conjoint. Thus, apart from the conjoint infections among the enteric parasites, helminth and coccidia, helminths lice, helminth and fleas, lice and fleas, lice and mites, lice and coccidia, fleas and mites, and fleas and coccidia had prevalent rates of 22.90, 7.46, 14.86, 12.16, 2.70, 6.76, 4.05, 8.11 per cent, respectively.

## 4. Discussion

The result of this study showed a wide range of parasitic infestations among village chickens in the study area. The prevalence of ectoparasites was high out of which lice infestation was outstanding. This result is in agreement with earlier studies [13, 14, 30–33] in America, South Africa and Nigeria. Following lice is flea with *Echinophaga gallinacea* as the most predominant species. The fact that fleas leave their host between meals [28] accounts for the predominance of stick *Echinophaga gallinacae* as the commonest flea type. Moreover, it is expected that the prevalence may have been higher as other flea types may have left the host after feeding and during overnight caging of the chicks. Fleas have been reported as the dominant ectoparasites by [14] while [33] showed that they were the least occurring of the ectoparasites. Moreover, [13] encountered no flea in their survey of blood and ectoparasites of domestic fowls in Ibadan, Western Nigeria. We speculate that these variations in result could be attributed to the season, time of the day, and the study location with respect to urban, periurban or pure village setting. With respect to mites, our results agreed with those of [13, 33] who listed mites as one of the common ectoparasites of village chicken.

 Ticks were apparently absent in our sample population. Earlier studies had demonstrated *Haemophysallis hoodi hoodi* as the only tick species among village chicken population in Nsukka area [13, 14, 34]. They however stated that tick infestation was not widespread. Our thorough examination of cracks and crevices within the sleeping areas of the chicks did not yielded any positive result showing the rarity of this parasite among this class of chicken. 

Ectoparasites, fleas, lice, and mites cause anaemia and depending on the degree of infestation may lead to egg abandonment in brooding hens. They also cause chick mortality attributed to starvation and immune depression under heavy infestation. This highlights the economic importance of ectoparasites in village chickens in the study area and Nigeria in general.

Adult chickens may have had higher prevalence due their gregariousness relative to growers and chicks thus exposing themselves more than the former. Also growers and chicks are still lack thorough knowledge of their environments and as a result shuttle less distances. Thus, they have close prevalence. We cannot advance reasons as to the variation in the prevalence of the various ectoparasites beyond the fact that this may be habitat related. 

The result on the prevalence of endoparasites calls for urgent attention to their prevention and control. Prevalence of 71% may account for major productivity losses such as mortalities, reduced growth, and reduced size at maturity, poor egg lay and feed efficiency, the common clinical features of these parasites [17, 35]. We were told during the survey that the highest losses occurred during the chick hood period and we strongly speculate that this may be associated with endoparasitism, especially coccidiosis in otherwise naïve chicks with under developed immune system. Earlier studies in the same ecological area as our study also demonstrated high prevalence of coccidiosis [36–39]. However, studies carried out in Kenya [5] showed that coccidiosis was not common among village chickens and suggested that it is a problem more related to intensive rather than extensive management. Moreover, the higher prevalence of all the parasites among the adults without apparent effect is indicative of adaptive response that enables them to act as carriers. We speculate that these adults may have undergone some kind of natural selection, a situation that gives the general impression of village chickens being more resistant to diseases than the exotics just as there local breeds of cattle that are resistant to some local disease conditions. However, our result may have been influenced by the season during which the survey was done [28, 33]. 

Helminth parasites are prevalent, although not as ubiquitous as has been reported in Ghana and Tanzania [18, 40]. The prevalence of the various helminth species in our study agrees with those of earlier investigations [41, 42] in their study involving domestic and grey breasted helmet guinea fowl in Nigeria. Helminths infestations are known to cause interference with host metabolism resulting in poor feed utilization and reduced growth rate as well as size and age at maturity [43]. These are known common characteristics of village chickens. The presence of the cestode, *Davainea proglotina* is also noteworthy because of its association with haemorrhagic enteritis which could complicate anaemia of ectoparasite origin. There were also many cases of mixed helminth infestations. The presence of *Heterakis gallinae* also poses the danger of enhanced transmission of *Histomonas meleagridis* to both susceptible turkeys and other poultry through shedding of the eggs in the environment. 

The concurrent infestations with two or more parasites, especially those with gastrointestinal predilection, heighten their role in early chick mortality and other productivity losses among the adults. This is particularly true of conjoint infestations with helminthes and coccidia whose combined effects on host metabolism could be devastating. Also, the conjoint infestation between the common ectoparasites and the enteric ones may add stress on the hosts with the attendant pathology.

In conclusion, this study demonstrated high prevalence of both ecto- and endoparasites among village chickens within the survey period and ecological zone. Based on the known pathologic effects of these parasites, the result of this study highlights both the eminent and potential constraints of these parasites to the overall village chicken production. We therefore recommend the institution of a programmed control measure for improved harnessing of the potentials of village chicken production in this region.

## Figures and Tables

**Table 1:** Age distribution of ectoparasite infestation in the sample population.

Age	No. sampled	No. positive	% infestation
Chicks	468	156	33.3
Growers	207	159	76.3
Adults	363	336	92.6
Total	1038	427	140.9

**Table 2:** Prevalence of ectoparasite species in the population, *n* = 427.

Parasite spp.	No. positive for specific ectoparasite.	% prevalence for population	% prevalence of parasite spp
Lice	261	25.14	62.15
Fleas	150	14.45	35.71
Mites	9	.87	2.14

**Table 3:** The prevalence of the various helminth species in the sample population, *n* = 261.

Helminth type	Population size	No. positive in the population	No. positive for individual helminth	Prevalence in the population	Prevalence among helminth types
*Ascaridia. galli*	261	93	45	17.2	48.39
*Heterakis gallinarum*	261	93	33	12.6	35.48
*Capilaria. spp*	261	93	15	5.7	16.13
*Raillietina spp*	261	93	15	5.7	16.13
*Syngamus treachea*	261	93	12	4.6	12.9
*Davainea proglottina*	261	93	9	3.45	9.68
*Sublura brumpti*	261	93	6	2.3	6.45
*Amoebataenia spp*	261	93	3	1.15	3.23
